# Chemotherapy following immune checkpoint inhibitors in recurrent or metastatic head and neck squamous cell carcinoma: clinical effectiveness and influence of inflammatory and nutritional factors

**DOI:** 10.1007/s12672-023-00774-4

**Published:** 2023-08-29

**Authors:** Akihiro Sakai, Koji Ebisumoto, Hiroaki Iijima, Mayu Yamauchi, Takanobu Teramura, Aritomo Yamazaki, Takane Watanabe, Toshihide Inagi, Daisuke Maki, Kenji Okami

**Affiliations:** Department of Otolaryngology, Head and Neck Surgery, Tokai University, School of Medicine, Isehara, Japan

**Keywords:** Recurrent, Distant metastasis, Head and neck cancer, Immune checkpoint inhibitor, Chemotherapy, Platelet to lymphocyte ratio

## Abstract

**Objective:**

This study aimed to evaluate the clinical effectiveness of chemotherapy following immune checkpoint inhibitors (ICI). The association between inflammatory and nutritional factors and prognosis has also been investigated.

**Methods:**

We retrospectively reviewed the medical records of recurrent or metastatic head and neck squamous cell carcinoma (RMHNSCC) patients who received chemotherapy following ICI therapy. The response rate and survival after chemotherapy, and nutritional and inflammatory factors, were examined.

**Results:**

The ICI before chemotherapy was nivolumab in 36 patients (70.6%) and pembrolizumab in 15 patients (29.4%). The chemotherapy regimens consisted of PTX in 32 patients (62.7%), PTX + Cmab in 9 (17.6%), and S1 in 10 (19.6%). The median overall survival (OS) was 20 months (95% CI 12–25), the estimated 12-month OS rate was 63.3%, the median progression-free survival (PFS) was 5 months (CI 4–6), and the 12-month PFS estimate was 8.9%. Univariate analysis significantly correlated Neutrophil-to-Lymphocyte Ratio (NLR), platelet-to-lymphocyte ratio (PLR), controlling nutritional status score (CONUT), and prognostic nutrition index (PNI) with OS and PFS. Additionally, these factors were significantly correlated with OS and PFS in the log-rank tests.

**Conclusions:**

Chemotherapy following ICI is highly effective. There were no significant differences in the chemotherapy regimens. Inflammatory and nutritional factors may associate with patient prognosis after chemotherapy.

## Introduction

Various chemotherapy regimens have been used to treat recurrent and recurrent or metastatic head and neck squamous cell carcinoma (RMHNSCC), but their prognosis remains poor [1–6]. However, treatment with a recently approved immune checkpoint inhibitor (ICI) has significantly prolonged the prognosis of RMHNSCC patients compared to conventional chemotherapy [7, 8]. In addition, adverse events were less severe than chemotherapy [9, 10], and treatment was possible while maintaining the patient's quality of life. Therefore, ICI has become widely used as the first-choice treatment for RMHNSCC. However, the response rates in the Checkmate 141 [7] and Keynote 048 [8] trials were 13.3% and 16.9% (pembrolizumab alone), respectively, and chemotherapy was the subsequent treatment of choice for nonresponders. Chemotherapy for patients with disease progression after ICI treatment is effective, and regimens combining platinum, cetuximab (Cmab), paclitaxel (PTX), and tegafur–gimeracil–oteracil potassium (S1) have been reported to date [11–16]. However, it is currently unclear which regimen is most effective in patients with RMHNSCC. Furthermore, several inflammatory and nutritional factors have been reported to be associated with prognosis [17–24]. However, few studies have examined the association with the efficacy or prognosis of chemotherapy in RMHNSCC [25]. The neutrophil-to-lymphocyte ratio (NLR) is a well-known prognostic factor for head and neck cancer [26]. Furthermore, various factors that can be easily assessed through blood tests, including the platelet to lymphocyte ratio (PLR) [27, 28], lymphocyte to monocyte ratio (LMR) [29], systemic immune-inflammation index (SII) [30, 31], C-reactive protein (CRP) to albumin ratio (CAR) [32, 33], controlling nutritional status (CONUT) score [18, 34], prognostic nutrition index (PNI) [23, 35], prognostic index (PI) [36], and the Glasgow Prognostic Score (GPS) [17], have been identified as valuable indicators for predicting the prognosis of individuals undergoing treatment for head and neck cancer [27, 28, 32, 33, 37]. However, it is unclear whether they are helpful in subsequent chemotherapy following ICI for RMHNSCC.

This study aimed to evaluate the clinical effectiveness of chemotherapy following ICI therapy. In addition, the effectiveness and response rates of each regimen were examined. Moreover, we investigated whether inflammatory and nutritional factors were associated with the effectiveness of chemotherapy following ICI in RMHNSCC, and also examined their correlation with the prognosis.

## Materials and methods

### Patient and data collection

We retrospectively reviewed the medical records of RMHNSCC patients who received ICI at Tokai University Hospital in Kanagawa, Japan, from April 2017 to June 2022 and those who received chemotherapy after receiving ICI. Patients were included if they had an Eastern Cooperative Oncology Group (ECOG) performance status of (0–2), had received at least one cycle of immunotherapy, had adequate hematologic, hepatic, pulmonary and renal functions, and could be imaged or clinically evaluated after chemotherapy.

Nivolumab was administered to patients every 2 weeks at 3 mg/kg or 240 mg/body doses. Pembrolizumab was administered at a dose of 200 mg every 3 weeks. After disease progression, chemotherapy with PTX, paclitaxel + cetuximab (PTX + Cmab), or S1 was administered.

PTX was administered at 100 mg/m^2^ once a week for 3 weeks, followed by a 1-week rest period. PTX + Cmab was initially administered with Cmab at 400 mg/m^2^ and 250 mg/m^2^ weekly. PTX was administered once weekly at a dose of 80 mg/m^2^, and S1 was administered at 80–120 mg/body/day, depending on the body surface area. S1 was administered for 2 weeks, followed by a 1-week rest period. Chemotherapy was selected and was decided at our multidisciplinary head and neck cancer conference based on each patient's condition. As a standard procedure, PTX or S1 was selected for patients with a history of Cmab use or pulmonary complications. S1 was also prescribed for patients over 80 years old and those who preferred outpatient treatment with oral therapy. For other cases, the combination of PTX and Cmab was utilized. Chemotherapy was continued until disease progression or unacceptable toxicity was observed, and the patients were followed up until death or the cutoff date (June 30, 2022).

The Institutional Review Board of Tokai University Hospital (20R248 and 22R223) approved this study, which was conducted according to the principles of the Declaration of Helsinki. Furthermore, the requirement for informed consent was waived because this study was a retrospective analysis of existing administrative and clinical data.

The clinical response to treatment was assessed every 4–12 weeks using computed tomography (CT). Tumor response was evaluated according to (Response Evaluation Criteria in Solid Tumors) version 1.1. The objective response rate (ORR) was defined as the percentage of patients who achieved a complete response (CR) or partial response (PR) as the best response. The disease control rate (DCR) was defined as the percentage of patients with CR, PR, or stable disease (SD) with the best response.

Overall survival (OS) was defined as the time from the start of treatment to the date of death or cutoff, regardless of the cause. Progression-free survival (PFS) was defined as the time from the beginning of treatment to the cutoff date when disease progression, death from any reason, or progression was no longer observed. The duration of response was defined as the time from the initial response (SD, PR, PD) to disease progression. Adverse events (AEs) were recorded using the National Cancer Institute Common Terminology Criteria for Adverse Events version (4.0).

### Definitions of inflammatory and nutritional factors

Albumin (Alb), C-reactive protein (CRP), and total cholesterol levels in serum and leukocytes, including neutrophils, lymphocytes, and monocytes, and platelet counts in peripheral blood were determined by blood analysis before starting chemotherapy. Based on the results, the values of following parameters were calculated: LMR, the ratio of lymphocyte count to monocyte count; NLR, the ratio of neutrophil count to lymphocyte count; PLR, the ratio of platelet count to lymphocyte count; CAR, the ratio of serum CRP level to serum Alb level; CONUT score, calculated using the serum Alb level, total lymphocyte count, and total cholesterol level; GPS, measured using the serum CRP level and serum Alb level: A CRP level > 1.0 mg/dL and Alb level < 3.5 g/dL were given a score of 2, a CRP level > 1.0 mg/dL or Alb level < 3.5 g/dL was given a score of 1, and a CRP level ≤ 1.0 mg/dL and Alb level ≥ 3.5 g/dL were given a score of 0.; PNI, which is 10 × serum Alb level + 0.005 × total lymphocyte count; and PI, measured using the serum CRP level and white bllod cell (WBC) count: PI 0 for CRP 1 mg/dL or less and WBC 11,000/μL or less, PI 1 if one of the two markers was elevated, and PI 2 if both markers were elevated.

### Statistical analysis

OS and PFS were estimated using the Kaplan–Meier method and evaluated using the log-rank test. The association between ORR, DCR, and subsequent chemotherapy groups was tested using the Kruskal–Wallis test for comparison of the three groups. Cutoff values for nutritional and inflammatory factors were determined by referring to ROC curves and were classified into two groups (high and low). The association between ORR, DCR, and each factor was assessed using the univariate logistic regression model. A Cox regression model analyzed the relationship between nutritional and inflammatory factors and OS or PFS. Logistic regression model and Cox regression model analyses were performed using EZR (Saitama Medical Center, Jichi Medical University, Japan), and other statistical analyses were performed using GraphPad Prism 8 software (GraphPad Software Inc., San Diego, CA, USA). Statistical significance was set at p < 0.05.

## Results

### Patient characteristics

From June 2017 to June 2022, 51 of the 110 RMHNSCC patients treated with ICI were eligible for treatment evaluation after chemotherapy following ICI. Their characteristics are summarized in Table 1. There were 48 males and three females with a median age of 66 years (range: 47–83 years). In 24 cases, the primary site was the hypopharynx, the oropharynx in 11 cases, and the larynx in 7 cases. The ECOG was 0–1 in almost all cases. The disease sites evaluated were locoregional recurrence in 32 cases and distant metastases in 19 cases. Regarding the line of chemotherapy, 43 cases received chemotherapy as second-line treatment after ICI, 7 cases for 3rd, and 1 for 4th. The type of ICI before chemotherapy was nivolumab in 36 patients and pembrolizumab in 15 patients. Regarding prior cetuximab treatment before ICI, seven patients received it, and 44 patients had never received it. The chemotherapy regimens consisted of PTX in 32 cases (62.7%), PTX + Cmab in 9 cases (17.6%), and S1 in 10 cases (19.6%).

**Table 1:** Patient characteristics

Variables	All		PTX		PTX + Cmab		S1	
	n	%	n	%	n	%	n	%
Sex								
Male	48	94.1	31	96.9	8	88.9	9	90.0
Female	3	5.9	1	3.1	1	11.1	1	10.0
Median age (range)	66 (47–83)		65 (49–77)		62 (51–74)		69 (47–83)	
Primary site								
Oral	4	7.8	2	6.3	0	0.0	2	20.0
Nasopharynx	1	2.0	1	3.1	0	0.0	0	0.0
Oropharynx	11	21.6	7	21.9	2	22.2	2	20.0
Hypopharynx	24	47.1	18	56.3	1	11.1	5	50.0
Larynx	7	13.7	4	12.5	2	22.2	1	10.0
Others	4	7.8	0	0.0	4	44.4	0	0.0
ECOG performance status								
0 or 1	50	98.0	31	96.9	9	100.0	9	90.0
> 2	1	2.0	0	0.0	0	0.0	1	10.0
Type of recurrence								
Locoregional	32	62.7	20	62.5	6	66.7	6	60.0
Distant	19	37.3	12	37.5	3	33.3	4	40.0
Chemotherapy line								
2nd	43	84.3	26	81.3	9	100.0	8	80.0
3rd	7	13.7	6	18.8	0	0.0	1	10.0
4th	1	2.0	0	0.0	0	0.0	1	10.0
Previous treatment								
Nivolumab	36	70.6	26	81.3	4	44.4	6	60.0
Pembrolizumab	15	29.4	6	18.8	5	55.6	4	40.0
Prior cetuximab treatment								
Yes	7	13.7	4	12.5	2	22.2	1	10.0
No	44	86.3	27	84.4	7	77.8	9	90.0

PTX: paclitaxel; Cmab: cetuximab; S1: tegafur–gimeracil–oteracil potassium; ECOG: Eastern Cooperative Oncology Group

### Effectiveness of subsequent chemotherapy

The ORR for patients after chemotherapy following ICI was 71.2% (37/51), and the DCR was 84.6% (44/51) (Table 2). The ORR by chemotherapy was 78.1% (25/32 patients), 88.9% (8/9 patients), and 40.0% (4/10 patients) for PTX, PTX + Cmab, and S1, respectively. DCR was 87.5% (28/32), 100% (9/9), and 70.0% (7/10). There was no significant difference in the ORR, but there was a significant difference in the ORR among the three groups.

**Table 2:** Effectiveness of subsequent chemotherapy

Variables	All	PTX	PTX + Cmab	S1	p value
Number of patients (%)	51	32 (62.7)	9	10	
Best response (%)					
Complete response	4 (7.84)	4 (12.5)	0 (0)	0 (0)	
Partial response	33 (64.7)	21 (65.6)	8 (88.9)	4 (40.0)	
Stable disease	7 (21.6)	3 (9.4)	1 (11.1)	3 (30.0)	
Progressive disease	7 (21.6)	4 (12.5)	0 (0)	3 (30.0)	
ORR (%)	71.2	78.1	88.9	40	0.03
DCR (%)	84.6	87.5	100	70	0.20

PTX, paclitaxel; Cmab, cetuximab; S1, tegafur–gimeracil–oteracil potassium; ORR, objective response rate; DCR, disease control rate

**Fig. 1:**
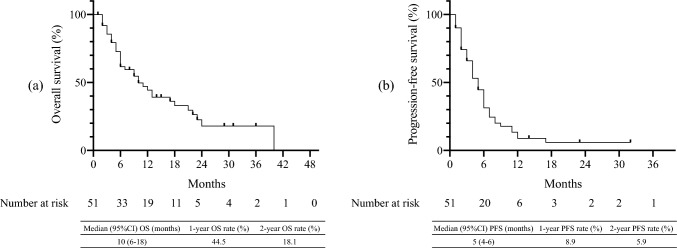
Kaplan–Meier curves of overall survival (**a**) and progression-free survival (**b**) for patients after chemotherapy following immune checkpoint inhibitor therapy. The median OS was 10 months (95% CI 6–18), and the estimated 12-month OS rate was 44.5% (**a**). The median PFS was 5 months (95% CI 4–6), and the 12-month PFS estimate was 8.9%

**Fig. 2:**
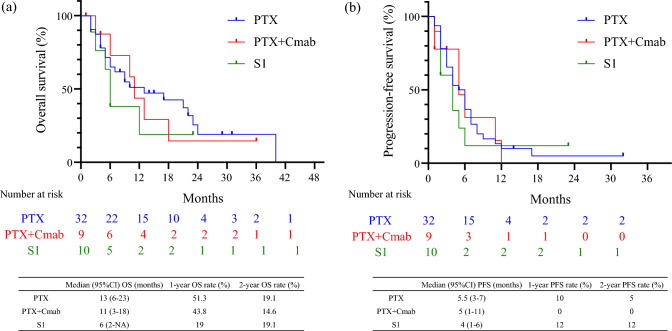
The overall survival (**a**) and the progression free survival (**b**) for patients administered PTX, PTX + Cmab, and S1. No significant differences in OS and PFS were observed between the chemotherapy groups

Figure 1 shows the estimated OS (Fig. 1a) and PFS (Fig. 1b) after the first dose of chemotherapy with ICI. The median OS was 10 months (95% CI 6–18), and the estimated 12-month OS rate was 44.5% (Fig. 1a). The median PFS was 5 months (95% CI 4–6), and the 12-month PFS estimate was 8.9%. The OS after chemotherapy (Fig. 2a) and PFS (Fig. 2b) are shown. No significant differences in OS and PFS were observed between the chemotherapy groups.

### Adverse event

The adverse events observed during chemotherapy are listed in Table 3. The most common AE was peripheral neuropathy in 6 patients (11.5%). The most common was thyroid dysfunction, pneumonia, liver dysfunction, and skin toxicity. Grade 3 or higher adverse events were observed in 10 patients (19.6%), and one died of interstitial pneumonia.

**Table 3:** Adverse events

Adverse events	All	Grade 1	Grade 2	Grade 3	Grade 4	Grade 5
Periferal neuropathy	6 (11.5)	1	4	1		
Thyroid dysfunction	5 (9.6)	2	3			
Pneumonia	4 (7.7)	1		1	1	1
Liver dysfunction	4 (7.7)		2	1	1	
Skin toxicity	4 (7.7)	2	1	1		
Diabetes mellitus	2 (3.8)			2		
Adrenal insufficiency	1 (1.9)			1		
Stomatitis	1 (1.9)	1				
Hypomagnesia	1 (1.9)	1	0			

### Prognostic analysis of patients who received chemotherapy following immune checkpoint inhibitors therapy

ORR, DCR and nutritional and inflammatory factors were analyzed for chemotherapy after ICI therapy (Table 4). Regarding the CUTOFF values (area under the curve) of each factor, BMI Cutoff: 20 (0.665), albumin cutoff: 3.5 (0.654), CRP Cutoff: 1.2 (0.672), LMR Cutoff: 2.5 (0.710), NLR Cutoff: 4.5 (0.659), PLR Cutoff: 268 (0.683), CAR Cutoff: 0.58 (0.678), CONUT Cutoff: 4 (0.703), and PNI Cutoff: 40 (0.715). No significant correlation was found between ORR or DCR and each factor. However, there was a trend for ORR to be associated with Age (odds ratio (OR) = 0.30, 95% confidence interval (CI) 0.08–1.09, P = 0.07) and for DCR to be associated with the NLR (OR = 6.57, 95% CI 0.73–59.2, P = 0.09) and PLR (OR = 4.37, 95% CI 0.76–25.2, P = 0.09). Table 5 shows the results of the Cox regression analysis between nutritional and inflammatory factors and OS and PFS in patients after chemotherapy. Examination of OS and nutritional and inflammatory factors showed significant correlations with Alb (hazard ratio (HR) = 3.00, 95% CI 1.49–6.04, P = 0.002), NLR (HR = 0.40, 95% CI 0.20–0.83, P = 0.013), PLR (HR = 0.26, 95% CI 0.12–0.55, P < 0.001), CAR (HR = 0.41, 95% CI 0.21–0.83, P = 0.012), CONUT score (HR = 0.25, 95% CI 0.12–0.52, P < 0.001), and PNI (HR = 4.32, 95% CI 2.03–9.16, P < 0.001). In addition, there was a significant correlation between PFS and NLR (HR = 0.53, 95% CI 0.28–0.98, P = 0.044), PLR (HR = 0.46, 95% CI 0.25–0.87, P = 0.016), CONUT score (HR = 0.50, 95% CI 0.26–0.95, P = 0.035), and PNI (HR = 2.36, 95% CI 1.27–4.38, P = 0.006).

**Table 4:** Prognostic analysis of objective response rate and disease control rate in patients who received chemotherapy following immune checkpoint inhibitor therapy

Variables	n	ORR (%)	Odds ratio	95% CI	p-value	DCR (%)	Odds ratio	95% CI	p-value
Age									
< 70	30	80.0	0.301	0.0833–1.09	0.067^†^	86.7	0.519	0.103–2.61	0.426
> 70	21	61.9				85.7			
Sex									
Male	48	75.0	6	0.499–72.2	0.16	87.5	3.5	0.274–44.8	0.335
Female	3	33.3				66.7			
BMI									
BMI < 20	21	76.2	1.37	0.384–4.89	0.63	85.7	0.923	0.184–4.63	0.923
BMI > 20	30	70.0				86.7			
Alb									
ALB < 3.5	22	81.8	2.37	0.628–8.93	0.20	90.9	2.08	0.364–11.9	0.409
ALB > 3.5	29	65.5				82.8			
CRP									
CRP < 1.2	26	73.1	1.06	0.308–3.61	0.93	88.5	1.46	0.292–7.3	0.645
CRP > 1.2	25	72.0				84.0			
LMR									
LMR < 2.5	26	69.2	0.711	0.206–2.45	0.59	80.8	0.365	0.0639–2.09	0.257
LMR > 2.5	25	76.0				92.0			
NLR									
NLR < 4.5	24	83.3	2.94	0.78–11.1	0.11	95.8	6.57	0.73–59.2	0.0931^†^
NLR > 4.5	27	63.0				77.8			
PLR									
PLR < 268	30	80.0	2.46	0.701–8.64	0.16	93.3	4.37	0.759–25.2	0.0985^†^
PLR > 268	21	61.9				76.2			
CAR									
CAR < 0.58	30	73.3	1.1	0.317–3.82	0.88	90.0	2.12	0.421–10.6	0.363
CAR > 0.58	21	71.4				81.0			
CONUT									
CONUT < 4	34	67.6	0.448	0.106–1.89	0.27	85.3	0.773	0.134–4.47	0.774
CONUT > 4	17	82.4				88.2			
GPS									
GPS 0	23	73.9	0.882	0.255–3.05	0.84	87.0	0.9	0.18–4.5	0.898
GPS 1,2	28	71.4				85.7			
Prognostic index									
PI 0	25	72.0	1.06	0.308–3.61	0.93	88.0	0.75	0.15–3.75	0.726
PI 1,2	26	73.1				84.6			
PNI									
PNI < 40	23	69.6	0.762	0.222–2.61	0.67	82.6	0.57	0.114–2.86	0.494
PNI > 40	28	75.0				89.3			

ORR: objective response rate; DCR: disease control rate; CI: confidence interval; BMI: body mass index; Alb: albumin; CRP: C-reactive protein; LMR: lymphocyte-to-monocyte ratio; NLR: neutrophil-to-lymphocyte ratio; PLR: platelet to lymphocyte ratio; CAR: CRP to albumin ratio; CONUT: controlling nutritional status; GPS: Glasgow Prognostic Score; PI: prognostic index; PNI: prognostic nutrition index

^†^p  < 0.01

**Table 5:** The univariate analysis of nutritional and inflammatory factors associated with overall survival and progression-free survival after chemotherapy

	OS			PFS		
	Hazard ratio	95% CI	p-value	Hazard ratio	95% CI	p-value
Age (< 70 or > 70)	1.018	0.5125–2.021	0.96	1.031	0.5684–1.872	0.919
BMI (Cutoff: 20)	1.885	0.9258–3.838	0.081	1.204	0.6577–2.205	0.547
Alb (Cutoff: 3.5)	3	1.491–6.038	**0.002**	1.457	0.7962–2.666	0.222
CRP (Cutoff: 1.2)	0.5825	0.2931–1.158	0.123	0.7539	0.4153–1.369	0.353
LMR (Cutoff: 2.5)	1.772	0.8836–3.552	0.107	1.358	0.7487–2.463	0.314
NLR (Cutoff: 4.5)	0.4018	0.1953–0.8268	**0.013**	0.5271	0.2829–0.9821	**0.044**
PLR (Cutoff: 268)	0.2555	0.1178–0.5543	**< 0.001**	0.4644	0.2491–0.8657	0.016
CAR (Cutoff: 0.58)	0.4146	0.2081–0.8258	**0.012**	0.554	0.3045–1.008	0.053
conut (Cutoff: 4)	0.2479	0.1191–0.5159	**< 0.001**	0.4998	0.2621–0.9528	**0.035**
GPS (0,1 or 2)	2.61	1.306–5.217	**0.007**	1.507	0.8148–2.787	0.191
Prognostic.index (0 or 1,2)	1.53	0.7704–3.039	0.224	1.29	0.7123–2.338	0.4
Propiostic.nutrition.index (Cutoff: 40)	4.315	2.032–9.164	**< 0.001**	2.361	1.272–4.384	**0.006**

Bold indicates statistically significant p-values

OS: overall survival; PFS: progression-free survival; CI: confidence interval; BMI: body mass index; Alb: albumin; CRP: C-reactive protein; LMR: lymphocyte-to-monocyte ratio; NLR: neutrophil-to-lymphocyte ratio; PLR: platelet to lymphocyte ratio; CAR: CRP to albumin ratio; CONUT: controlling nutritional status; GPS: Glasgow Prognostic Score; PI: prognostic index; PNI: prognostic nutrition index

**Fig. 3:**
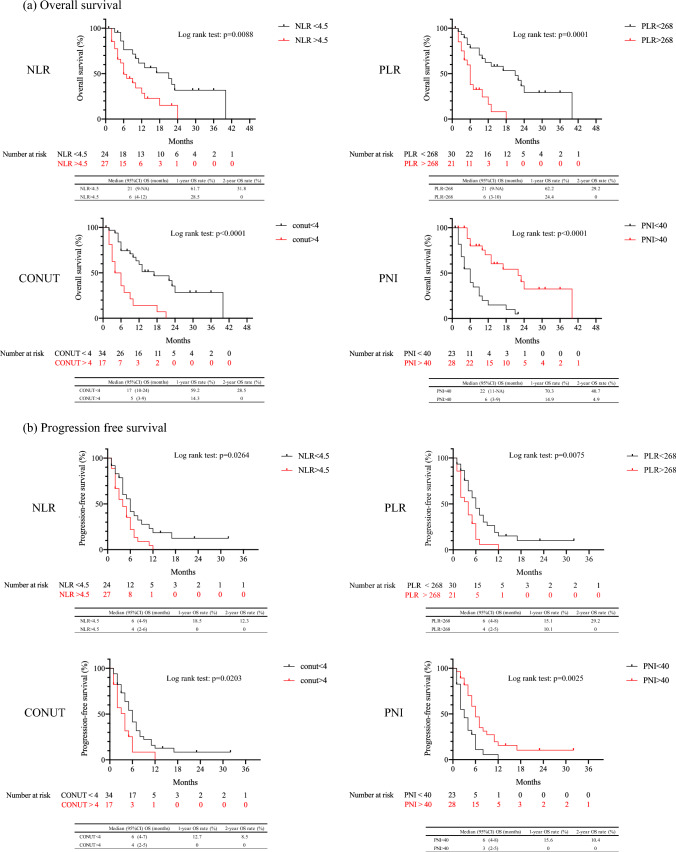
Kaplan–Meier curves of overall survival (**a**) and progression-free survival (**b**) for patients by nutritional and inflammatory factors divided by cutoff values. Significant differences in OS and PFS were observed between the two groups

Figure 3 shows the results of Kaplan–Meier survival curves and log-rank tests by cutoff values for NLR, PLR, CONUT score, and PNI, which were highly correlated with OS and PFS using the Cox regression model. Significant differences in OS and PFS were observed between the two groups for NLR (p = 0.0088, p = 0.264), PLR (p = 0.0001, p = 0.0075), CONUT score (p < 0.0001, p = 0.0203), and PNI (p < 0.0001, p = 0.0025).

## Discussion

Immunotherapy, a breakthrough treatment for head and neck cancer, has become more widely used, and treatment outcomes and prognoses are gradually becoming clearer [10, 15, 38]. In addition, many studies have reported relatively better results with chemotherapy after ICI compared to conventional chemotherapy for RMHNSCC [12, 13, 15, 39] Our department employs three regimens: PTX, PTX + Cmab, and S1, but the single agent, PTX regimen, is primarily used. However, this was because many patients had a history of cetuximab when nivolumab was started in our department, and we wanted to avoid duplication of cetuximab. After its initiation, the PTX regimen was clinically effective and had few adverse events; therefore, the PTX monotherapy regimen has been used as a general rule. In this study, the ORR and DCR for patients treated with chemotherapy following ICI were 71.2% and 84.6%, respectively, and the ORR after chemotherapy was 78.1%, 88.9%, and 40.0% for PTX, PTX + Cmab, and S1, respectively. The highest ORR for PTX + Cmab and lowest ORR for S1 were observed. PTX + Cmab had the best response rate but was not significantly different from that of PTX. In addition, a study on the estimated OS and PFS from the first dose of chemotherapy after ICI showed favorable results, with a median OS of 10 months (95% CI 6–18), an estimated 12-month OS rate of 44.5%, a median PFS of 5 months (95% CI 4–6), and an estimated 12-month PFS rate of 8.9%. Compared to reports of patients receiving PTX + Cmab [14], we found no significant difference in OS, although PFS was slightly lower. In addition, there were no significant differences among the three groups by regimen, similar to the results reported by Yasumatsu et al. [15]. Although the effects between regimens vary from report to report [12–15, 40–43], all reports indicate that chemotherapy after ICI is effective. Our results also showed that chemotherapy was effective regardless of regimen, and the results were comparable to previous reports [14, 15, 41]. Due to the small number of cases and some biases in our study, it was difficult to describe the differences in efficacy between regimens. However, as more patients accumulate, it is expected to become clearer which regimen is more effective.

Regarding safety, although there was a concern about an increase in adverse events with chemotherapy after using ICIs, this study showed no particular increase in adverse events. However, there was one case of serious interstitial pneumonia. Matsuo et al. stated that patients treated with nivolumab followed by Cmab-containing chemotherapy have a higher risk of drug-induced interstitial lung disease compared to other regimens [44]. In patients with documented pulmonary dysfunction, Cmab-containing chemotherapy should be administered with careful monitoring.

Although several factors may be associated with the prognosis or efficacy of chemotherapy after ICI in RMHNSCC, few studies have examined those factors [25]. Recently, nutritional and inflammatory factors have been reported to be associated with prognosis [17, 19, 21–25], and we investigated whether these factors were associated with prognosis after chemotherapy in RMHNSCC.

The results of this study showed no significant differences between the ORR or DCR and each factor. However, there was a trend suggesting that ORR might be associated with age, whereas DCR might be associated with NLR and PLR. Matsuki et al. reported that hematological inflammatory markers, specifically elevated NLR and modified GPS, were significantly associated with DCR, but not with ORR, in patients with RMHNSCC treated with nivolumab [45]. Our previous study found no significant correlation between inflammatory and nutritional factors and the ORR in patients receiving ICI therapy [46]. However, DCR showed a significant correlation with the systemic immune-inflammation index and a trend associated with other factors such as PLR. Clinical studies examining the association between ORR or DCR and survival after chemotherapy have reported that ORR is not associated with prognosis and that DCR is associated with prognosis [47]. These results suggest that inflammatory and nutritional factors may be associated with DCR rather than ORR, which may be correlated with long-term prognosis.

Furthermore, we examined the associations between these inflammatory and nutritional factors and prognosis. Univariate Cox regression analysis for OS and PFS by factor showed that Alb, NLR, PLR, CAR, CONUT, GPS, and PNI were significantly correlated with OS, and NLR, PLR, CONUT, and PNI were significantly correlated with PFS. Additionally, these four factors (NLR, PLR, CONUT score, and PNI) were significantly correlated with OS and PFS in the log-rank test.

And reflect prognosis, with better nutritional status and lower inflammatory status associated with better prognosis. Wakasaki et al. reported that CRP and NLR were prognostic factors for chemotherapy after nivolumab in RMHNSCC [25]. The NLR is a well-known biomarker that has been reported to be an independent prognostic factor in head and neck cancer [26]. The results of the present study, as well as previous reports, showed that NLR is associated with the prognosis of chemotherapy after ICI. However, to date, no other studies have examined the prognostic value of inflammatory and nutritional factors for chemotherapy after ICI, and the role of these factors remains unclear. Our results also demonstrated that PLR, CONUT score, and PNI were significantly associated with prognosis. PLR is defined as the platelet-to-lymphocyte ratio. Increased platelet counts have been implicated in increased cancer invasiveness and indirect interactions between cancer cells and platelets through secreted molecules, making them more aggressive [48]. In contrast, lymphocytes constitute a significant component of the host immune system and can eliminate cancer cells and prevent tumor progression [49]. In other words, relatively high platelet and low lymphocyte counts may predict a poor prognosis. Takenaka et al. [50] reported that PLR was associated with poor survival in patients with HNSCC. Additionally, PLR is reportedly associated with poor prognosis in various carcinomas [24, 51, 52]. These results showed that PLR was also a prognostic factor for the patients in ICI after chemotherapy.

The CONUT score is a nutritional scoring tool used to screen and identify hospitalized patients with malnutrition [34]. This score, calculated from serum albumin, total lymphocyte count, and total cholesterol, has recently emerged as a tool to assess the nutritional and immunological status of patients with malignancies [18, 21, 53]. The PNI, which is calculated based on the serum albumin concentration and peripheral blood lymphocyte count, is a nutritional scoring tool that is used to assess the nutritional and immune status of patients with cancer [19]. The PNI has been shown to be a useful prognostic tool in predicting survival and guiding treatment decisions in patients with various types of cancer [20, 23]. The CONUT score and PNI are indicators of nutritional status. However, Lin et al. demonstrated that the CONUT score has a superior prognostic value compared to the NLR, PLR, and LMR [18]. This finding further emphasizes the correlation between nutritional status, immune status, and prognosis. Notably, they also revealed that the prognostic value of the CONUT score was superior to that of the PNI score, suggesting an important role of cholesterol in determining the prognosis of tumors. Our results revealed no significant differences between the two factors. However, both factors were associated with prognosis, suggesting they could be useful tools for chemotherapy following ICI treatment.

In summary, our results showed that several inflammatory and nutritional factors are correlated with the prognosis of patients treated with chemotherapy after ICI. Although the usefulness of these factors differs among reports, they all indicate that inflammatory and nutritional factors are associated with cancer patient prognosis. In the future, these factors are expected to contribute to the prognosis of cancer patients by providing nutritional support at an early stage concurrently with cancer treatment.

This study had several limitations. First, this was a single-center retrospective study that evaluated a small number of patients. Second, the PTX + Cmab and S1 groups were small, making it difficult to assess the usefulness of the regimen and possibly causing bias in drug selection. Third, nutritional and inflammatory factors must be interpreted cautiously because of the many factors involved. Finally, randomized prospective trials are needed to optimize chemotherapy after ICI treatment for RMHNSCC.

## Conclusions

We evaluated the effectiveness of chemotherapy after ICI treatment in our department. The median OS was 20 months (95% CI 12–25), the estimated 12-month OS rate was 63.3%, the median PFS was 5 months (95% CI 4–6), and the 12-month PFS estimate was 8.9%, with favorable results. There were no significant differences in each chemotherapy regimen's effectiveness and response rates. Inflammatory and nutritional factors were examined in terms of OS and PFS. These factors may associate with patient prognosis of chemotherapy after ICI treatment.

## Data Availability

All relevant data has been presented in this manuscript.

## Abbreviations

ICI: Immune checkpoint inhibitors
PFS: Progression-free survival
PLR: Platelet-to-lymphocyte ratio
RMHNSCC: Recurrent and recurrent or metastatic head and neck squamous cell carcinoma
CT: Computed tomography
ORR: Objective response rate
DCR: Disease control rate
CI: Confidence interval
CR: Complete response
PR: Partial response
SD: Stable disease
OS: Overall survival
PFS: Progression-free survival
AE: Adverse event
BMI: Body mass index
Alb: Albumin
CRP: C-reactive protein
LMR: Lymphocyte-to-monocyte ratio
NLR: Neutrophil-to-lymphocyte ratio
PLR: Platelet to lymphocyte ratio
CAR: CRP to albumin ratio
CONUT: Controlling nutritional status
GPS: Glasgow Prognostic Score
PI: Prognostic index
PNI: Prognostic nutrition index
PTX: Paclitaxel
Cmab: Cetuximab
S1: Tegafur–gimeracil–oteracil potassium
ECOG: Eastern Cooperative Oncology Group
